# Modal energy transfer by thermally induced refractive index gratings in Yb-doped fibers

**DOI:** 10.1038/s41377-018-0061-6

**Published:** 2018-09-05

**Authors:** Christoph Stihler, Cesar Jauregui, Andreas Tünnermann, Jens Limpert

**Affiliations:** 1 Institute of Applied Physics, Abbe Center of Photonics, Friedrich-Schiller-Universität Jena, Albert-Einstein-Str. 15, 07745 Jena, Germany; 2 Helmholtz-Institute Jena, Fröbelstieg 3, 07743 Jena, Germany; 3 Fraunhofer Institute for Applied Optics and Precision Engineering, Albert-Einstein-Str. 7, 07745 Jena, Germany

## Abstract

Thermally induced refractive index gratings in Yb-doped fibers lead to transverse mode instability (TMI) above an average power threshold, which represents a severe problem for many applications. To obtain a deeper understanding of TMI, the evolution of the strength of the thermally induced refractive index grating with the average output power in a fiber amplifier is experimentally investigated for the first time. This investigation is performed by introducing a phase shift between the refractive index grating and modal interference pattern, which is obtained by applying a pump power variation to the fiber amplifier. It is demonstrated that the refractive index grating is sufficiently strong to enable modal energy coupling at powers that are significantly below the TMI threshold if the induced phase shift is sufficiently large. The experiments indicate that at higher powers, the refractive index grating becomes more sensitive to such phase shifts, which will ultimately trigger TMI. Furthermore, the experimental results demonstrate beam cleaning above the TMI threshold via the introduction of a positive phase shift. This finding paves the way for the development of a new class of mitigation strategies for TMI that are based on controlling the phase shift between the thermally induced refractive index grating and modal interference pattern.

## More power to fibre optic lasers

Researchers have cracked a ‘did the chicken come before the egg’ dilemma in fibre optic lasers. The new understanding of ‘transverse mode instability’ could lead to even stronger lasers. Jens Limpert and colleagues at Germany’s Friedrich-Schiller Universität Jena provided experimental evidence that increasing the power of an optic fibre beyond a certain point degrades laser output because interference patterns between light waves within the fibre core become ‘out of sync’ with the core’s refractive index grating; called a phase shift. Researchers had not been sure if the instability of light waves within the fiber core was initially triggered by this phase shift or by the growing core’s refractive index grating with increasing power. A poor understanding of transverse mode instability has halted further improvements in optic fibre technologies, which produce high power lasers for industry, medicine and defence. The new information could lead to strategies that mitigate the phenomenon.

## Introduction

Fiber laser technology has earned a solid reputation as a power-scalable laser concept with excellent beam quality. Currently, fiber lasers deliver the highest diffraction-limited average power of any solid-state lasers and, thus, have enabled a wide range of applications in industry, medicine, defense, and science^1^. However, the discovery of the phenomenon of transverse mode instabilities (TMI) in 2010^2,3^ has brought the evolution of the average output power of active fibers to a sudden halt. The TMI effect occurs once an average power is exceeded and manifests itself as a dynamic energy coupling between transverse modes. Since this leads to an abrupt degradation in the quality and stability of the output beam at high average output powers, the TMI phenomenon prevents the development of new fields of applications. Although several approaches to suppressing TMI have been proposed^4–15^, TMI is still the main limiting factor for these systems. Hence, to develop new effective mitigation strategies, it is important to obtain a deeper understanding of the phenomenon.

The physical origin of TMI is linked to the interference between two or more transverse modes in the active fiber. The resulting modal interference pattern (MIP) generates a quasi-periodic heat distribution along the fiber, which gives rise to a quasi-periodic temperature profile. Via the thermo-optic effect, this temperature profile is translated into a quasi-periodic refractive index pattern, namely, a thermally induced refractive index grating (RIG). Since this RIG has been produced by the interference between fiber modes, it has the correct features to potentially couple energy between them^16^. However, by itself, the RIG is not sufficient for such an energy transfer (ET) to take place. For this to happen, a phase shift between the RIG and the MIP is additionally necessary^17^. Finally, both requirements, namely, a strong RIG and a phase shift, must be fulfilled simultaneously to drive TMI. In this context, it is of substantial importance to determine what ultimately triggers TMI: the surpassing of a threshold strength of the RIG or the introduction of a sufficiently large phase shift. Although various models of TMI have been developed and refined over the years^18–26^, which deliver results that are supported by the findings of experimental studies^27–29^, the initial trigger of TMI has not been determined. Furthermore, even though there is a broad consensus that TMI is ultimately caused by an RIG, no experimental proof for the existence of the RIG has been presented until now. By clarifying these fundamental yet open questions about one of the most interesting but also damaging effects that have ever been observed in active fibers, this work can pave the way for the development of new mitigation strategies for TMI.

## Results

### Phase shift and modal energy transfer

Recently, it was shown that it is possible to wash out the RIG in the longitudinal direction by modulating the pump power^15^. This resulted in a reduction of the modal energy coupling at average powers above the TMI threshold. The operating principle of this washing out is described in detail in ref. ^15^. A pump modulation periodically stretches and compresses the MIP since the signal output power and, hence, the transverse temperature profile at each position along the fiber change. Thus, in turn, the transverse refractive index profile of the fiber is modified via the thermo-optic effect, which alters the local guiding properties of the fiber. This leads to a modification of the index differences between the transverse modes, which results in a change of the MIP^1^. Crucially, and essential to this work, this MIP-variation, in turn, leads to a phase shift between the MIP and the RIG because the RIG cannot follow the MIP changes instantaneously.

We have confirmed this behavior via simulations, which are based on the model that is presented in ref. ^15^, where the full 3D-resolved rate equations are solved with semi-analytic formulas that calculate the temporal responses of the 3D inversion pattern and the 3D temperature profile to changes in the system. Then, these profiles are used to obtain the MIP and the RIG along the fiber with a temporal resolution of 5 µs. To provide a clear illustration of the temporal evolution of this phase shift between the MIP and the RIG, an instantaneous pump power jump from 100 W to 200 W was applied to the system and the energy coupling between the fiber modes was switched off in the simulations. Thus, the purpose of the simulations is not to exactly predict the phase shift that will be obtained in the experiment, but to illustrate that a pump power variation can generate a phase shift. For this example, a 1 m long rod-type fiber with 80 µm core diameter (MFD ~ 65 µm), 228 µm pump-cladding diameter, 1.2 mm outer fiber diameter, a V-parameter of 7 and doped with 3.25 × 10^25^ Yb ions per m³ was simulated. These fiber parameters are close to those that were used in the experiments. The fiber was seeded by an average power of 5.5 W at 1030 nm (10% higher-order mode content) and counter-pumped at 976 nm. After the pump power jump, the pump power was kept constant at 200 W. In this scenario, the temporal evolutions of the MIP and the RIG were calculated over a temporal window of 2.5 ms.

**Fig. 1:**
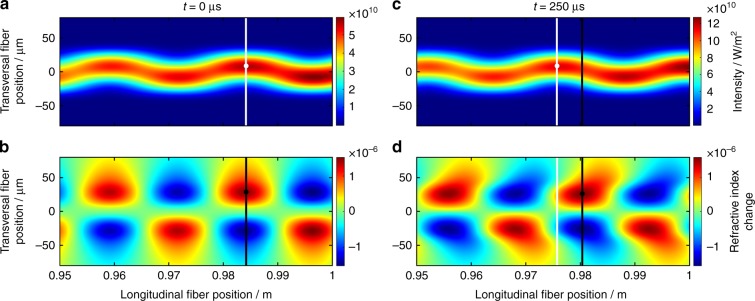
Phase-shift evolution that is caused by a pump power jump from 100 W to 200 W. **a** The modal interference pattern (last 50 mm) immediately prior to the pump power jump (*t* = 0 µs). The white line indicates the position of the intensity maximum of the MIP that will be tracked. **b** The refractive index grating (last 50 mm) immediately prior to the pump power jump (*t* = 0 µs). The black line indicates the position of the maximum of the RIG that is aligned with the tracked MIP maximum. **c** The modal interference pattern (last 50 mm) after the pump power jump (*t* = 250 µs). The white line indicates the new position of the tracked MIP maximum. **d** The refractive index grating (last 50 mm) after the pump power jump (*t* = 250 µs). The black line indicates the new position of the tracked RIG maximum

Figure 1 depicts excerpts from Supplementary Video 1 and illustrates the MIP (top graphs) and the radially anti-symmetric part of the refractive index profile, which corresponds to the RIG (bottom graphs) at the end of the fiber (last 50 mm) immediately before the pump power jump (Fig. 1a, b) and 250 µs after it (Fig. 1c, d). Immediately before the pump power jump (at *t* = 0 µs), the MIP (Fig. 1a), and the RIG (Fig. 1b) are in phase; hence, the maxima of the MIP are located at the same positions along the fiber as the maxima of the RIG. To visualize the phase shift (i.e., the difference in the positions of the MIP and RIG maxima), we have tracked the positions of one MIP maximum and one RIG maximum. The positions of these maxima are marked with white and black vertical lines, respectively. The temporal evolutions of the positions of these tracked maxima are shown in Supplementary Video 1. According to the video, with elapsing time and, thus, with increasing temperature in the fiber, the MIP is strongly compressed. This is because the length of each period of the MIP along the fiber decreases as the temperature increases due to the thermally induced waveguide changes, which lead to a larger difference between the effective refractive indices of the fundamental mode (FM) and the higher-order modes (HOMs)^19^. This compression of the MIP periods accumulates over the fiber length, i.e., the sum of all period changes that occur in the fiber before a specified position determines the shift of the MIP maximum at this position. Thus, after 250 µs, the marked maximum of the MIP (white line in Fig. 1) is shifted by 8 mm toward the seed side of the fiber (in contrast, an MIP maximum near the middle of the fiber is only shifted by 1 mm).

By compressing the MIP, the heat source for the RIG is shifted and, afterwards, a new RIG arises (in phase with the new MIP) while the former RIG starts to decay (out of phase with the new MIP). Our simulations demonstrate that the old RIG remains strong even after the MIP has been shifted by a significant distance (e.g., 8 mm after 250 µs). Thus, an effective RIG can be defined as the superposition of all the slightly shifted RIGs that are created along the fiber at various instants in time. The maximum of this effective RIG, which was in phase with the marked MIP maximum at *t* = 0 µs, is marked with a black line in Fig. 1c, d. Comparing the positions of the white and black lines in Fig. 1c, d, a significant phase shift has developed between the MIP and the effective RIG at the end of the fiber after 250 µs. At this time, the phase shift has reached its maximum of 4.6 mm at the end of the fiber, which corresponds to ~1.2 rad at a local period length of the MIP of 23.8 mm. From now on, as shown in Supplementary Video 1, the phase shift slowly decreases and asymptotically approaches zero since the compression of the MIP slows and the effective RIG adapts increasingly well to the new MIP. In this way, via our simulations, we have shown that when changing the pump power in a fiber laser system, a phase shift between the MIP and the RIG can be introduced. A detailed investigation of the development of the phase shift is beyond the scope of this article; it is discussed in detail in ref. ^30^. Based on this finding, we conclude that if the phase shift is sufficiently large and the RIG is sufficiently strong, the pump modulation will enable ET between the transverse modes.

**Fig. 2:**
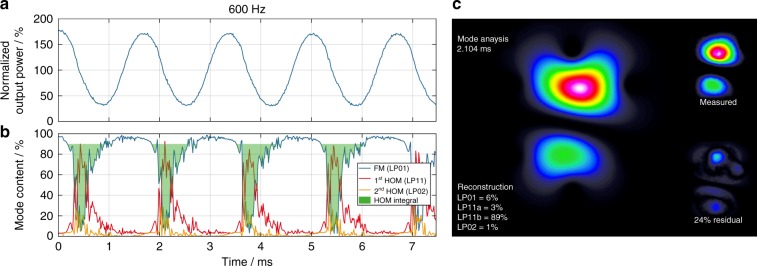
Negative energy transfer that is induced by the pump modulation above the TMI threshold, as analyzed via mode reconstruction of the high-speed camera frames. **a** The temporal evolution of the signal output power, which is normalized to the average signal power of 350 W, with a pump modulation with 600 Hz modulation frequency and ±75% (±262.5 W) modulation depth. **b** The temporal evolution of the relative modal content (FM (LP_01_) blue, 1st HOM (LP_11_) red, 2nd HOM (LP_02_) yellow); the HOM integral is shaded green. **c** The reconstructed intensity profile (on the left, including the relative modal contents of various transverse modes), measured intensity profile (top right) and residual intensity profile (bottom right) at the time of negative energy transfer during the pump modulation; excerpt from Supplementary Video 2

To investigate this behavior experimentally on a short time scale, the near-field image of the output beam of a ~1.1 m long Yb-doped large-pitch fiber (LPF)^31^ with an active core of ~65 µm and a TMI threshold of 233 W (measured according to ref. ^27^) that is seeded with 5 W at 1030 nm and counter-pumped at 976 nm was recorded with a high-speed camera with a framerate of up to 67,000 frames per s. Analyzing these data with a mode reconstruction algorithm (based on ref. ^28^ and detailed in the Materials and methods section), we observed ET from the FM to the HOMs at the falling edge of the sinusoidal pump modulation, which we refer to as negative ET. This negative ET is caused by a phase shift, which we will define as a negative phase shift, in which the MIP has a slightly longer period than the RIG. We have investigated the negative ET above the TMI threshold (at 350 W average output power) at a pump modulation frequency of 600 Hz and with a modulation depth of ±75% (±262.5 W) in the fiber amplifier set-up that is described above. The results of the mode reconstruction algorithm are illustrated in Fig. 2.

Figure 2a illustrates the temporal evolution of the signal power, which follows the sinusoidal pump modulation and is normalized to the average signal power. Arising from the mode reconstruction, the temporal evolution of the relative modal content is depicted on the same time scale in Fig. 2b. The negative ET occurs at the falling edge of the sinusoidal pump modulation, where the phase shift between the MIP and the RIG is negative. Figure 2c is an excerpt from Supplementary Video 2 and displays the beam intensity profile as measured with the high-speed camera (top right), the reconstructed beam intensity profile (on the left, including the relative modal contents of various transverse modes) and the residual intensity distribution (bottom right) at the time of negative ET with a modulation frequency of 600 Hz.

According to Fig. 2b, the mode reconstruction cannot reach an FM content of 100% due to the intrinsic accuracy of our mode reconstruction algorithm, which typically is in the range of 10% and is related to the residual intensity that could not be assigned to any of the simulated modes. Thus, a baseline of 90% for the FM content is used for the analysis of our measurements. Hence, we consider all regions with an FM content of greater than 90% as pure FM operation. The higher residual intensity at times of negative ET is due to the mode reconstruction algorithm taking only four modes into account (LP_01_, LP_11a_, LP_11b_, LP_02_). However, many more modes participate in such a process in the few-mode fiber (LPF) that is used in the experiments. To quantitatively measure the negative ET, we have calculated the integral of the HOM content, which is defined as the baseline value (90%) minus the integral of the FM content over one modulation period and normalized to the latter. The HOM integral is represented by the green-shaded area in Fig. 2b. Apart from the region with negative ET, according to Fig. 2b, the fiber emits light in pure FM operation most of the time, even though the average output power exceeds the TMI threshold. The first reason for this behavior is the washing out of the RIG, which reduces the energy coupling when the modulation frequency and depth are chosen appropriately^15^. The second reason for this behavior is the positive phase shift that is induced at the rising edge of the sinusoidal pump modulation due to the compression of the MIP (i.e., the MIP has a shorter period length than the RIG), as demonstrated in our simulations and shown in Fig. 1. Theoretically, such a positive phase shift generates ET from the HOMs toward the FM^17^. This beam-cleaning phenomenon will be discussed in detail later in this article.

**Fig. 3:**
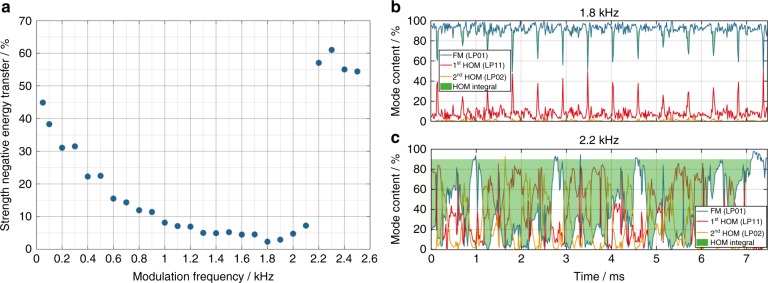
Dependence of the negative energy transfer on the modulation frequency above the TMI threshold. **a** The strength of the negative energy transfer (represented by the HOM integral) as a function of the modulation frequency at 350 W average output power and for ±75% (±262.5 W) pump modulation depth. **b** The temporal evolution of the relative modal content (FM (LP_01_) blue, 1st HOM (LP_11_) red, 2nd HOM (LP_02_) yellow) for a pump modulation frequency of 1.8 kHz; the HOM integral is shaded green. **c** The temporal evolution of the relative modal content (FM (LP_01_) blue, 1st HOM (LP_11_) red, 2nd HOM (LP_02_) yellow) for a pump modulation frequency of 2.2 kHz; the HOM integral is shaded green

Additionally, we have investigated the dependence of the strength of the negative ET on the modulation frequency, which is depicted in Fig. 3a. For this experiment, both the average output power (350 W) and the modulation depth (±75%, ±262.5 W) were kept constant. Even though a negative ET is induced by the pump modulation in all cases, it becomes weak for a broad frequency range. This indicates that the RIG is being washed out. The strongest weakening of the ET for our fiber occurs between 1.3 kHz and 2 kHz. However, this frequency range depends on the fiber parameters such as the mode field diameter, cladding design, and core composition. The negative ET for this frequency range is shown exemplarily for 1.8 kHz in Fig. 3b. Such high frequencies also demonstrate that the occurrence of the negative ET cannot be related to any build-up or decay time of the RIG. The minimum measured build-up time of TMI in ref. ^32^ was 1.6 ms for a fiber with 33 µm mode field diameter. Since the fiber that was used in our experiments has nearly twice the mode field diameter, the build-up time in our fiber is probably ~3 ms. When using a pump modulation with a frequency of, e.g., 2 kHz, the pump power increases for only 250 µs. The negative energy transfer occurs ~350 µs after the sine minimum, which is significantly below the TMI build-up time of 3 ms. Thus, this negative energy transfer must be related to the negative phase shift that is induced at the falling edge of the sinusoidal pump modulation and cannot be satisfactorily explained by the build-up and decay times of the RIG.

For frequencies below 1.3 kHz, the strength of the negative ET increases nonlinearly to 50 Hz for two reasons: first, the RIG can better adapt itself to the MIP changes; thus, the washing out is less effective. Second, the introduced phase shift increases because there is more time for the fiber core to change its temperature; thus, the MIP undergoes stronger compression and stretching. The first effect results in a stronger RIG and the second in a stronger negative ET.

Above 1.8 kHz, the RIG tends to become stronger again because the stretching and compression of the MIP become progressively faster, which makes it more complicated for the RIG to follow. This weakens the washing out of the RIG. The point at which the RIG is no longer washed out sufficiently is reached at 2.2 kHz, where the system abruptly becomes unstable and shows a significantly increased ET over a large portion of one modulation period. This behavior is shown in Fig. 3c. In summary, in addition to the dependence of the negative ET on the pump modulation frequency, our experiments have demonstrated that an ET is triggered by the introduction of a phase shift between the MIP and the RIG, which was induced and controlled by applying a pump modulation to a fiber amplifier.

### Energy transfer below the TMI threshold

As already mentioned, identifying the initial trigger of TMI is of substantial importance for the development of new mitigation strategies. One of the main theories on this trigger states that the RIG is sufficiently strong to enable energy coupling between the fiber modes, even below the TMI threshold; however, no significant phase shift exists to ultimately provoke the instabilities^1^. Consequently, the strength of the RIG grows further with increasing average output power until it becomes highly sensitive to even small phase shifts, which can be induced by, e.g., signal or pump power noise. Thus, this theory claims that a small noise-induced phase shift is sufficient to enable energy coupling between modes and trigger TMI if the sensitivity of the RIG is sufficiently high. This implies that ET between the fiber modes could take place below the TMI threshold if a sufficiently large phase shift between the RIG and the MIP is introduced.

**Fig. 4:**
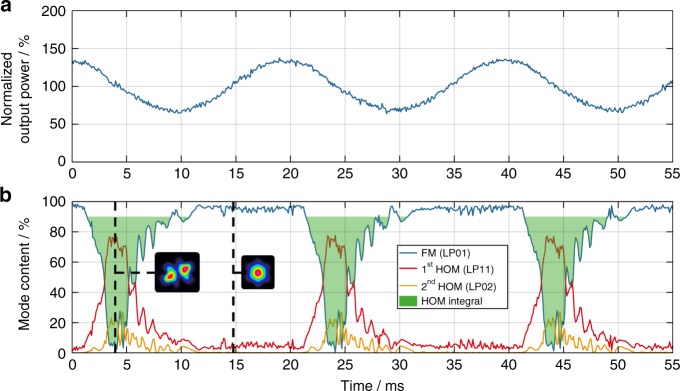
Negative energy transfer below the TMI threshold. **a** The temporal evolution of the signal output power, normalized to the average signal power of 150 W, with a pump modulation with 50 Hz modulation frequency and ±50 W modulation depth. **b** The temporal evolution of the relative modal content (FM (LP_01_) blue, 1st HOM (LP_11_) red, 2nd HOM (LP_02_) yellow) for 50 Hz pump modulation frequency; the HOM integral is shaded green; the insets are excerpts from Supplementary Video 3 and represent the beam profiles at the times of negative ET (left) and positive ET (right)

To answer the question about the initial trigger of TMI, we have investigated whether ET also occurs at powers that are below the TMI threshold. By applying the pump modulation technique at 150 W average output power (i.e., below the TMI threshold of 233 W), thereby introducing a phase shift, it was possible to observe ET significantly below the TMI threshold, as shown in Fig. 4. Figure 4a illustrates the temporal evolution of the signal output power, normalized to the average signal power, and Fig. 4b shows the reconstructed temporal evolution of the relative modal content, including the HOM integral (green shading) for a modulation frequency of 50 Hz and a modulation depth of ±50 W. Thus, it is ensured that the signal power remains below the TMI threshold at all times. A modulation frequency of 50 Hz was chosen to enable a strong ET (Fig. 3a) and, thus, simplify the analysis. The insets of Fig. 4b are excerpts from Supplementary Video 3 and represent the beam profiles at the times of negative ET (left) and positive ET (right). According to the experimental observation of ET below the TMI threshold, the initial trigger for TMI is most likely a noise-induced phase shift between the RIG and the MIP, which supports the theory that is described in ref. ^1^.

### Energy transfer as a function of the pump modulation frequency below the TMI threshold

**Fig. 5:**
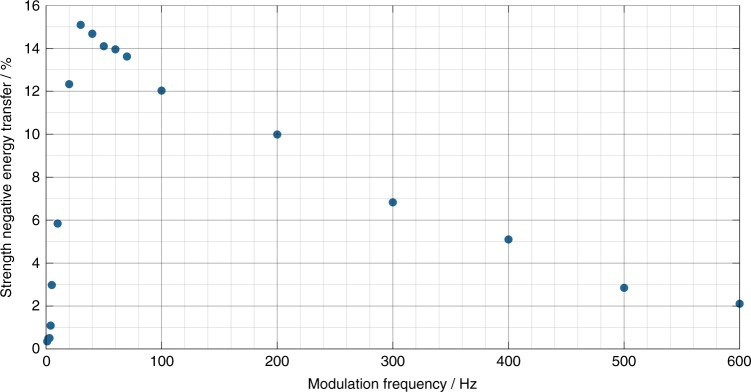
Strength of the negative energy transfer (represented by the HOM integral) as a function of the pump modulation frequency below the TMI threshold (at 150 W average output power and ±50 W modulation depth)

In addition to the experiments whose results are displayed in Fig. 3, we have investigated the dependence of the strength of the negative ET on the modulation frequency below the TMI threshold. By varying this parameter, the induced maximum phase shift is changed, which directly influences the strength of the negative ET. The experiment was performed at an average output power of 150 W (i.e., below the TMI threshold) with a modulation depth of ±50 W. According to Fig. 5, when the modulation frequency is zero, no ET occurs because no phase shift between MIP and RIG is introduced. However, a steep increase in the strength of the negative ET occurs when a slow pump modulation is applied. This is due to the increasingly long temporal delay (i.e., phase shift) that is induced between the RIG and the MIP. For the fiber that is tested in these experiments and for the given average output power and modulation depth, the strength of the negative ET reaches its maximum at ~30 Hz and decreases slowly as the frequency increases further. This behavior can be explained by the shorter time during which the fiber core changes its temperature at higher modulation frequencies. In turn, this leads to weaker compression and stretching of the MIP and, thus, to a smaller phase shift. At very high modulation frequencies, when the phase shift is too small to enable any significant negative ET with the RIG strength at the given output power, the curve in Fig. 5 will reach zero. Moreover, the washing out of the RIG reduces its strength; thus, the negative ET is further reduced. The frequency at which the zero-ET level is reached and the frequency of the maximum negative ET depend on the average output power of the fiber laser system, among others. With a rising average output power, the frequency of the maximum strength of the negative ET increases because less time is needed to change the temperature in the mode area, which shrinks due to the higher heat load in the fiber. Furthermore, the frequency at which the zero-ET level is reached will also increase when the output power increases because the RIG is stronger; thus, a smaller phase shift will suffice to enable ET. When the average power exceeds the TMI threshold, a behavior that is similar to that depicted in Fig. 3a is observed.

### Analysis of the grating growth below the TMI threshold

To investigate the growth of the RIG strength, we have analyzed the negative ET at various average output powers with the HOM integral that is described above. To facilitate comparison of the results, we must ensure that the introduced phase shift is constant over all average powers. This was guaranteed by enforcing a constant change in the period of the MIP by applying the same modulation frequency (50 Hz) and the same absolute modulation depth (±50 W) across all measurements. Again, we have chosen a modulation frequency of 50 Hz to obtain a strong ET to simplify the analysis.

**Fig. 6:**
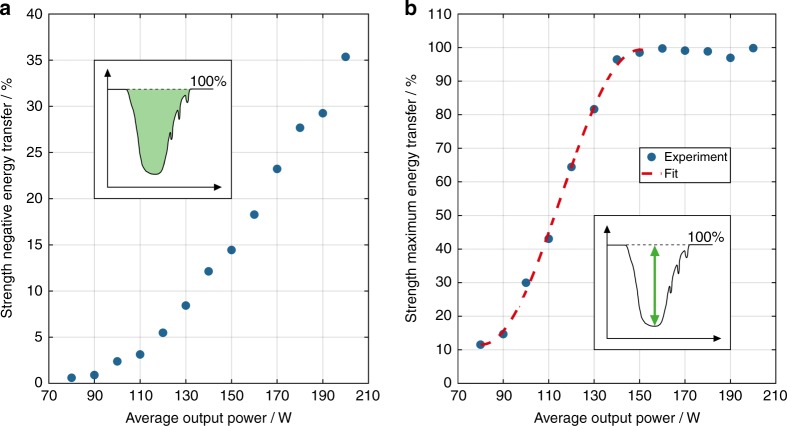
Strength of the negative energy transfer as a function of the average output power below the TMI threshold. **a** The strength of the negative energy transfer below the TMI threshold, represented by the HOM integral as a function of the average output power with a pump modulation with 50 Hz modulation frequency and ±50 W modulation depth; the inset represents a schematic diagram of the calculated HOM integral (green-shaded area). **b** The evolution of the strength of the maximum negative energy transfer as a function of the average output power, including experimental data (blue dots) and a fit curve according to Eq. (1) (red dashed line); the inset shows a schematic diagram of the calculated maximum negative ET (green arrow)

The dependence of the strength of the negative ET (represented by the HOM integral) on the average output power below the TMI threshold is shown in Fig. 6a. The strength of the negative ET depends nonlinearly on the average power. For 190 W and above, the strength of the negative ET is underestimated because the calculated HOM integral decreases due to a strong back transfer from the HOM into the FM, which manifests itself as dips in the HOM content. The reason for this phenomenon is that the phase shift between the RIG and the MIP becomes larger than π at various locations along the fiber, which corresponds to a positive phase shift that enables an energy coupling from the HOM to the FM.

Additionally, we have investigated the evolution of the strength of the maximum negative ET, i.e., the HOM content at the time at which the strongest negative ET occurs, as a function of the average power, which is represented by the blue dots in Fig. 6b. The maximum possible energy coupling, which corresponds to a full ET from the FM into the HOMs, is reached at ~150 W average output power, where the curve saturates. Derived from coupled-mode theory and according to ref. ^33^, the maximum coupling strength, which is denoted as *Κ*_max_, for transmission gratings is expressed as

1 $${{K}}_{\rm {max}} = {{\rm {sin}}}^2\left( {\kappa \cdot L} \right)$$

where *κ* is the coupling coefficient and *L* is the length of the grating. This behavior should also be observable for the RIG in our fiber, even though it is non-uniform over the whole fiber length due to the anisotropy that is induced by the power extraction under counter-pumping conditions. However, it is possible to define an equivalent uniform RIG that will result in the same maximum possible energy coupling^33^. Thus, we have fitted the experimental data of Fig. 6b up to the saturation level of 150 W, which is the region where Eq. (1) is valid, with the following function:

2 $$c_1 \cdot {\rm {sin}}^2\left[ {\left( {P_{\rm {out}} + c_2} \right) \cdot L_{\rm {F}} \cdot c_3} \right] + c_4$$

The fit coefficients are used to adjust the amplitude (*c*_1_ = 87.76%), the x-offset (*c*_2_ = −80.48 W), the period (*c*_3_ = 0.02045 (W m)^−1^), and the y-offset (*c*_4_ = 11.57%) of the sin^2^ function. *L*_F_ is also a constant and represents the length of the tested fiber (*L*_F_ = 1.1 m). *P*_out_ is the average output power, which represents the measured data and is directly proportional to *κ*. This is because *P*_out_ determines the generated heat and, thus, the change in the refractive index, which finally defines *κ* (ref. ^6,33^). Details about the fit function and its parameters are provided in the methods section. The fit matches the experimental data very well as the curve of the maximum negative ET rises with a sin^2^ behavior. Hence, the RIG strength (*κ* × *L*) must increase linearly with the average output power *P*_out_. This is the first time that it has been possible to experimentally characterize the growth of an RIG in a high-power fiber amplifier and demonstrate the linear dependence of the grating strength on the average output power. Thus, the experiments have demonstrated that at average output powers that exceed the TMI threshold, the RIG has attained a strength that is significantly higher than the necessary strength for initiating energy coupling. This makes the RIG highly sensitive to even small phase shifts, which most likely suffice to trigger TMI. Such small phase shifts can be induced by, e.g., signal or pump power noise^1^.

### Beam cleaning above the TMI threshold

**Fig. 7:**
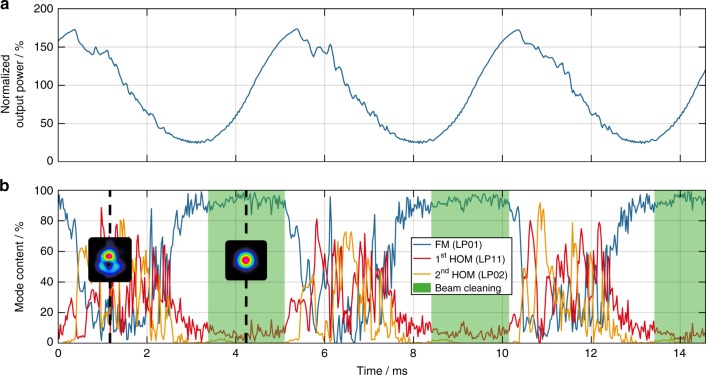
Beam cleaning above the TMI threshold. **a** The temporal evolution of the signal output power, normalized to the average signal power of 350 W, with a pump modulation with 200 Hz modulation frequency and ±75% (±262.5 W) modulation depth. **b** The temporal evolution of the relative modal content (FM (LP_01_) blue, 1st HOM (LP_11_) red, 2nd HOM (LP_02_) yellow); the beam-cleaning area is shaded green; the insets are excerpts from Supplementary Video 4 and represent the beam profiles at the times of negative ET (left) and positive ET/beam cleaning (right)

As mentioned previously, in addition to a negative phase shift, a positive phase shift is induced by the pump modulation when the MIP is compressed. This positive phase shift leads to a positive ET with the energy flowing from the HOMs toward the FM. We will refer to this effect as beam cleaning in the following. Beam cleaning occurs at any average output power, either below or above the TMI threshold, if an RIG exists and a phase shift between it and the MIP can be induced. Figure 7 illustrates this behavior at 350 W average output power (i.e., significantly above the TMI threshold) with a modulation frequency of 200 Hz (far away from the optimal frequency for washing the RIG out, as shown in Fig. 3a) and a modulation depth of ±75% (±262.5 W). Figure 7a shows the temporal evolution of the signal output power, normalized to the average signal power, and Fig. 7b shows the reconstructed temporal evolution of the relative modal content, including at the time of beam cleaning (shaded green). The insets of Fig. 7b are excerpts from Supplementary Video 4 and represent the beam profiles at the times of negative ET (left) and beam cleaning (right).

According to Fig. 7, the introduction of a positive phase shift during the rising edge of the pump leads to a pure FM operation, which is maintained while the phase shift is larger than zero and smaller than *π*. Thus, we hypothesize that a sustained positive phase shift would lead to a stable FM operation of the fiber laser system. This finding is the first experimental proof of positive energy coupling and it paves the way for the development of a new class of mitigation strategies for TMI, of which a key feature is the control of the introduced phase shift. This is particularly promising because we confirmed that such a beam cleaning occurs even when the pump modulation is such that the signal power exceeds the TMI threshold at all times. Additionally, the beam-cleaning effect is not limited to any special fiber type since the only requirement is an existing RIG.

## Discussion

In this article, we have experimentally proven the existence of a thermally induced refractive index grating in active fibers under a high thermal load, which leads to modal energy transfer. This RIG is responsible for TMI, which is currently the main limiting factor for the further power scaling of fiber laser systems with nearly diffraction-limited beam quality. By introducing a phase shift between the MIP and the RIG, we demonstrated that the latter is sufficiently strong to couple energy between transverse modes at powers that are significantly below the TMI threshold. Hence, most likely the initial trigger for TMI is the phase shift between the MIP and the RIG, not the RIG reaching sufficiently high strength to initiate ET. Furthermore, it was demonstrated that the strength of the negative ET depends nonlinearly on the average output power, whereas the RIG strength increases linearly with the average output power, which is consistent with theory. Finally, experimental proof was presented for the existence of a positive energy coupling from the HOMs to the FM when there is a positive phase shift. This beam-cleaning effect is not limited to any average power region or special fiber type since it requires only an existing RIG. Consequently, this finding will enable the development of a new class of mitigation strategies for TMI, of which a key feature is the control of the introduced phase shift.

## Materials and methods

### Experimental set-up

The fiber that was used for the presented experiments is a ~1.1 m long Yb-doped large-pitch fiber (LPF)^31^ with an active core of ~65 µm that was seeded by a 5 W signal at 1030 nm and counter-pumped by a 976 nm laser diode that could deliver up to 2 kW. This pump diode was connected to a driver that was modulated with up to 3 kHz. A function generator that was connected to the driver was used to create a sine wave with the desired modulation frequency and depth. In the free-running fiber laser system (without pump modulation), a TMI threshold of 233 W was measured via the method that is detailed in ref. ^27^. The amplified output beam was guided into an analysis path that was composed of a thermal power meter, high-speed camera, and two photodiodes. The first photodiode was calibrated to the power meter and detected fast changes of the output power of the beam, which was completely focused on the photodiode. This photodiode also measured the modulation frequency and modulation depth when the pump modulation was applied. The second photodiode (stability photodiode) had a small detection area and a rise time of 1 ns. The beam was imaged onto this stability photodiode, whereby the beam size was kept larger than the detection area, similar to the set-up that was described in ref. ^27^. Thus, the photodiode recognized a change in the beam intensity profile at the fiber output from a FM-like beam to a HOM-like beam as a drop in the photocurrent and a change from a HOM-like beam to a FM-like beam as an increase in the photocurrent. The high-speed camera, namely, “Phantom v611”, recorded the near-field image of the beam and detected fast changes in the modal content with a framerate of up to 67,000 frames per second. The frames of the high-speed camera were analyzed via a mode reconstruction algorithm that outputs a quantitative measure of the modal content and, thus, the energy coupling.

### Investigations on the modal interference pattern

**Fig. 8:**
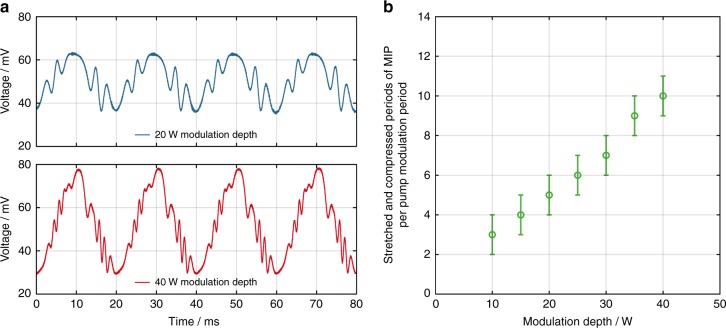
Demonstration of the stretching and compression of the modal interference pattern during the pump modulation. **a** The signals of the stability photodiode during a pump modulation at 50 W average output power with a modulation frequency of 50 Hz and modulation depths (peak to valley) of 20 W (top graph, blue) and 40 W (bottom graph, red). **b** The number of periods of the MIP that are compressed and stretched during a single period of the pump modulation as a function of the modulation depth (peak to valley)

The periodic stretching and compression of the MIP along the fiber are visible as periodic spatial variations of the beam intensity profile at the fiber output. This experimentally demonstrates the presence of the MIP, which ultimately gives rise to an RIG. The change in the MIP was measured by imaging the output beam of the fiber amplifier on the stability photodiode. Figure 8a depicts the photodiode signal at an average output power of 50 W, which is significantly below the TMI threshold. The pump was modulated with 50 Hz and modulation depths that ranged from 10 to 40 W (from peak to valley). The periodic oscillations on top of the sine waves in Fig. 8a represent the compression and stretching of the MIP. These result in a change of the beam intensity profile at the output of the fiber, which, in turn, leads to a change of the intensity profile on the stability photodiode and, thus, to a variation in the detected photocurrent. This demonstrates that there is an MIP and strongly suggests the existence of an RIG. Furthermore, from Fig. 8b, we infer that the number of observed oscillations, i.e., the stretched and compressed periods of the MIP at the end of the fiber, during a single period of the pump modulation increases in the measured range nearly linearly with the modulation depth. Hence, the displacement of the MIP at the end of the fiber increases linearly with the modulation depth.

### Mode reconstruction

The beam that is measured with the high-speed camera is decomposed into the different transverse fiber modes via an algorithm that is based on the algorithm that is described in ref. ^28^. In principle, a multidimensional optimization algorithm searches for the coherent combination of modes that best matches the measured beam intensity profile by varying the mode phases and intensities. We automated this algorithm in terms of mode-size scaling, mode rotation and heat load search. For this, the mode set of the experimental fiber is simulated for a specified heat load and the simulated modes are, at first, incoherently superposed. To simplify the mode reconstruction, we limited the modes in this superposition to LP_01_, LP_11a_, LP_11b_, and LP_02_. The incoherently superposed simulated modes are scaled and rotated to optimize the overlap with the measured beam profile. Afterwards, all measured beam profiles are normalized to the maximum intensity of each frame to avoid problems in the reconstruction with the changing power. Then, the scaled and rotated simulated modes are coherently superposed and their phases and intensities are changed until the best match for one of the measured beam profiles is found. This process is repeated for selected high-speed camera frames. To determine the accuracy of the modal reconstruction, the measured beam intensity profile and the reconstructed one are subtracted from each other to obtain a residual intensity profile, which could not be assigned to any of the simulated modes. This procedure is repeated for various heat loads and the heat load with the lowest residual intensity is selected. Finally, all frames are reconstructed with the calculated heat load, scaling factor and rotation angle.

The temporal evolution of the relative modal content is plotted after the mode reconstruction, which provides the opportunity to quantitatively measure a possible ET. Furthermore, the intensity of each high-speed camera frame is integrated and normalized to the average intensity of all frames. The temporal evolution of this integrated power resembles a sine wave when a sinusoidal pump modulation is applied. Thus, the modulation frequency and the modulation depth can be obtained from this graph. Additionally, for visualization purposes, a video is created that is comprised of the reconstructed beam, along with the measured beam and the residual intensity pattern of each frame (Supplementary Videos 2, 3 and 4).

### Fit function for the strength of the maximum negative energy transfer

To fit the measured strength of the maximum negative ET of the RIG in the tested fiber amplifier, we adapted Eq. (1), which represents the maximum coupling strength, namely, *Κ*_max_, for transmission gratings^33^. This resulted in Eq. (2), which was used to fit the measured data. The fit function contains one variable, namely, the measured average output power, which is denoted as *P*_out_ and is directly proportional to the coupling strength *κ* of the RIG. The reason for this is that *P*_out_ defines the generated heat and, thus, the change in the refractive index, which determines *κ* (ref. ^6,33^). Furthermore, the constant *L*_F_ of the fit function is the length of the tested fiber, which is 1.1 m, and represents the length of the grating, which is denoted as *L*, in Eq. (1). Additionally, four fit coefficients are used to adjust the amplitude (*c*_1_ = 87.76%), the x-offset (*c*_2_ = −80.48 W), the period (*c*_3_ = 0.02045 (W m)^−1^), and the y-offset (*c*_4_ = 11.57%) of the sin^2^ function. In the following, we will discuss the need for these four fit coefficients. Coefficients *c*_1_ (amplitude) and *c*_4_ (y-offset) are required due to the accuracy of the mode reconstruction algorithm, which typically is in the range of 10% and is related to the residual intensity that could not be assigned to any of the simulated modes. Thus, a modal ET that is below 10% cannot be measured with this method. The need for *c*_2_ (x-offset) can be explained by the sensitivity of our measurement: Since we applied a pump modulation depth of ±50 W, we are only able to measure above a theoretical limit of 50 W average output power. This is because at an average output power of 50 W, there will be no radiation in the minimum of the sinusoidal pump modulation (*P*_out_ = 0 W) that can be recorded by the high-speed camera. In practice, this sensitivity limit is higher (~80 W) because of noise in the high-speed-camera frames at low powers, which leads to errors in the mode reconstruction. Finally, *c*_3_ (period) is needed to adjust the slope of the sin^2^ function (i.e., the period), which is determined by the generated heat per watt of average output power. The more heat that is generated, the steeper the curve is, and the shorter the period of the sin^2^ function will be.

### Data availability

The data that support the plots within this paper and other findings of this study are available from the corresponding author upon reasonable request.

## Electronic supplementary material

Phase-shift evolution caused by a pump power jump from 100 W to 200 W

Energy transfer above the TMI threshold induced by the pump modulation (mode reconstruction of the high-speed camera frames)

Energy transfer below the TMI threshold induced by the pump modulation (mode reconstruction of the high-speed camera frames)

Beam cleaning above the TMI threshold (mode reconstruction of the high-speed camera frames)
